# Endoscopic nasal multilevel skull base reconstruction combined with lumbar cistern drainage in the treatment of infection caused by cerebrospinal fluid leakage: A case report

**DOI:** 10.1097/MD.0000000000040556

**Published:** 2024-11-29

**Authors:** Longshan Zhou, Xiaoqian Yang, Jiatao Lv, Zonglei Chong

**Affiliations:** a Department of Neurosurgery, The Second People’s Hospital of Liaocheng, Liaocheng, Shandong, China; b Department of Neurology, Liaocheng People’s Hospital, Liaocheng, Shandong, China; c Department of Neurosurgery, Liaocheng People’s Hospital, Liaocheng, Shandong, China.

**Keywords:** central nervous system infection, cerebrospinal fluid leakage, lumbar cistern drainage, multi-level skull base reconstruction, neuroendoscopic transnasal surgery

## Abstract

**Rationale:**

Traumatic cerebrospinal fluid leakage can cause complications such as meningitis, ventriculitis and brain abscess. It is necessary to formulate individualized treatment strategies, such as the use of antibiotics and skull base reconstruction methods. The application of nasal septum mucosal flap can significantly reduce the incidence of cerebrospinal fluid leakage. Neuroendoscope has been widely used in the treatment of skull base diseases, and the application of continuous lumbar cistern drainage technology has effectively controlled cerebrospinal fluid leakage. This paper introduces a case of long-term cerebrospinal fluid leakage complicated with infection. Neuroendoscopic multilevel skull base reconstruction combined with lumbar cistern drainage has achieved good clinical results, and this combined treatment is rarely reported in the literature. In order to promote the surgical effect of cerebrospinal fluid leakage, this combined treatment scheme is introduced here.

**Patient concerns:**

A 43-year-old male was admitted with headache for 7 days, aggravation with fever and vomiting for 2 days.

**Diagnoses:**

Cerebrospinal fluid culture suggests central nervous system infection, history and symptoms suggest traumatic cerebrospinal fluid rhinorrhea, and computed tomography (CT) shows old skull base fracture.

**Interventions:**

Intracranial infection was controlled after anti-infection treatment with sensitive antibacterial drug ceftriaxone sodium. Repair of cerebrospinal fluid rhinorrhea by transsphenoidal endoscope. Continuous lumbar cistern drainage (LCD) was performed after operation to promote the healing of the leak.

**Outcomes:**

After comprehensive treatment, the patient did not have CSF rhinorrhea again and was discharged.

**Lessons:**

Antibiotics combined with endoscopic multi-segment skull base reconstruction and lumbar cistern drainage are effective in treating infection caused by cerebrospinal fluid leakage.

## 1. Introduction

Traumatic cerebrospinal fluid (CSF) leakage is mainly caused by traffic injury, falling injury from a height and hitting injury, and the incidence rate is 1% to 3%, which can cause complications such as meningitis, ventriculitis, brain abscess, subdural hematoma, tension pneumocranium, etc, and will endanger the life of patients in severe cases.^[1]^ Hyper-pneumatized petrous bone and paranasal sinuses can be attributed as a risk factor for formation of spontaneous CSF leaks.^[2]^ Craniocerebral trauma leads to skull base fracture involving the dura mater and arachnoid structure of skull base, which further leads to CSF flowing to the nasal cavity, middle ear, orbit and surrounding sinus, forming CSF rhinorrhea, otorrhea or orbital leakage.^[3]^ Most traumatic CSF leakage occurs in the early stage of injury, about half of patients occur within 2 days after injury, about 70% patients occur within 1 week after injury, and almost all patients occur within 3 months after injury.^[4]^ Although most patients with traumatic CSF leakage can be effectively treated after diagnosis, the prognosis of some complicated patients is still not ideal.^[5]^ Especially when CSF leakage is combined with intracranial infection or complicated craniocerebral trauma, the prognosis of patients is often poor, so it is necessary to formulate individualized treatment strategies, such as the scheme and duration of non-surgical treatment, the timing and mode of surgical treatment, and the use and duration of antibiotics.^[6]^ Various skull base reconstruction methods and multi-layer skull base reconstruction techniques have achieved different degrees of success.^[7]^ Safe, firm and effective skull base reconstruction is very important. At present, there is no uniform standard for skull base reconstruction, and most scholars emphasize soft skull base reconstruction.^[8]^ The nasoseptal flap is an excellent anterior skull base reconstructive technique. Patients with high-flow intraoperative CSF leaks had a 94% successful reconstruction rate.^[9]^ In recent years, neuroendoscope has been widely used in the treatment of skull base diseases.^[10]^ Especially for patients with high-flow CSF leakage with open third ventricle and extensive cistern during operation.^[11]^ The application of Gasket-seal technique, pedicled nasal septum mucosal flap, in situ bone flap technique and continuous lumbar cistern drainage (LCD) effectively controlled CSF leakage.^[12]^ On September 4, a case of CSF leakage was treated in the Department of Neurosurgery, Second People’s Hospital of Liaocheng City. The case is characterized by long-term cerebrospinal fluid leakage complicated with infection. Neuroendoscopic multi-level skull base reconstruction combined with lumbar cistern drainage has achieved good clinical results, and this kind of combined treatment is rarely reported in the literature. In order to promote the surgical effect of cerebrospinal fluid leakage, this combined treatment scheme is introduced here, and the summary report is as follows.

## 2. Case presentation

**Figure 1.**
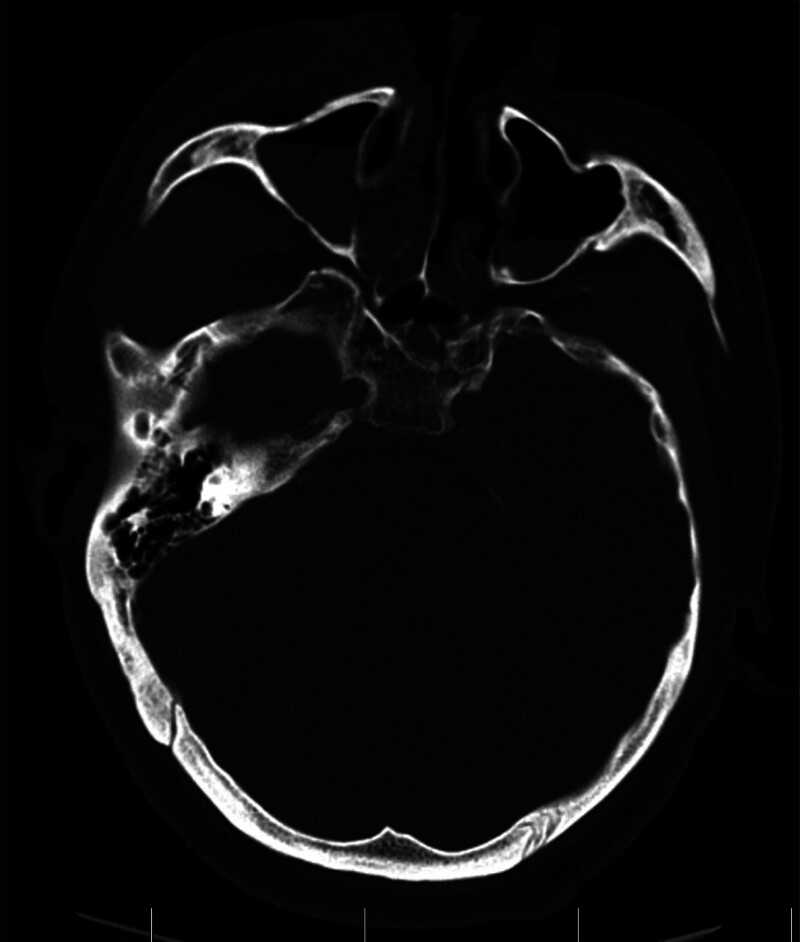
On August 2nd, 1 year ago, CT images of the patient showed fracture lines in the frontal bone, maxilla and sphenoid bone, which involved the inner wall of the right orbit and showed high-density shadows in the sinus cavity. CT = computed tomography.

The patient, a 43-year-old male, was admitted to the hospital on September 4, complaining of “headache for 7 days, aggravation with fever and vomiting for 2 days.” The patient started with headache symptoms, such as swelling and pain in the top of the head and occipital region. At first, the patient didn’t care and didn’t receive treatment. Later, the headache symptoms worsened, and the pain was obvious when lying down and coughing, accompanied by fever. The highest body temperature was 38.5°C, accompanied by nausea and vomiting. Infusion of “acyclovir, mannitol” in the local hospital was not effective, so the patient came to our hospital for further treatment. Past medical history: The patient was treated in our hospital 1 year ago because of head injury caused by falling from a height. After trauma, brain computed tomography (CT) showed multiple fractures of frontal bone, maxilla and sphenoid bone, hematocele in sinus cavity, pneumatosis under skin and swelling of soft tissue in forehead and face (Fig. 1). This case has no symptoms related to the disease before, and there is no similar disease in the family. Physical examination: clear mind, poor spirit, neck resistance, Brudzinsk sign and Kernig sign positive. According to clinical symptoms, signs and examination, the initial diagnosis is: central nervous system infection, traumatic CSF rhinorrhea, old skull base fracture.

**Figure 2.**
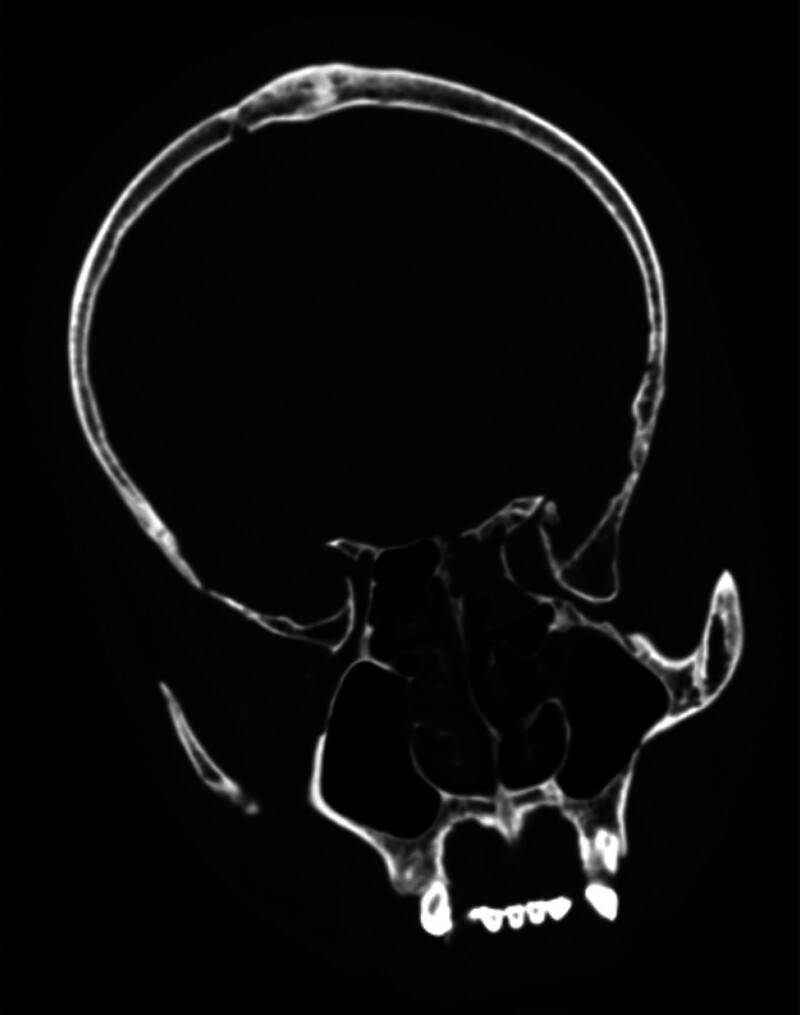
On the CT image of the patient on September 11th, the localization examination of CSF rhinorrhea showed that the bone on the top wall of the right sphenoid sinus was discontinuous, and there were localized bone defects and liquid low-density shadows in the right sphenoid sinus. CSF = cerebrospinal fluid, CT = computed tomography.

**Figure 3.**
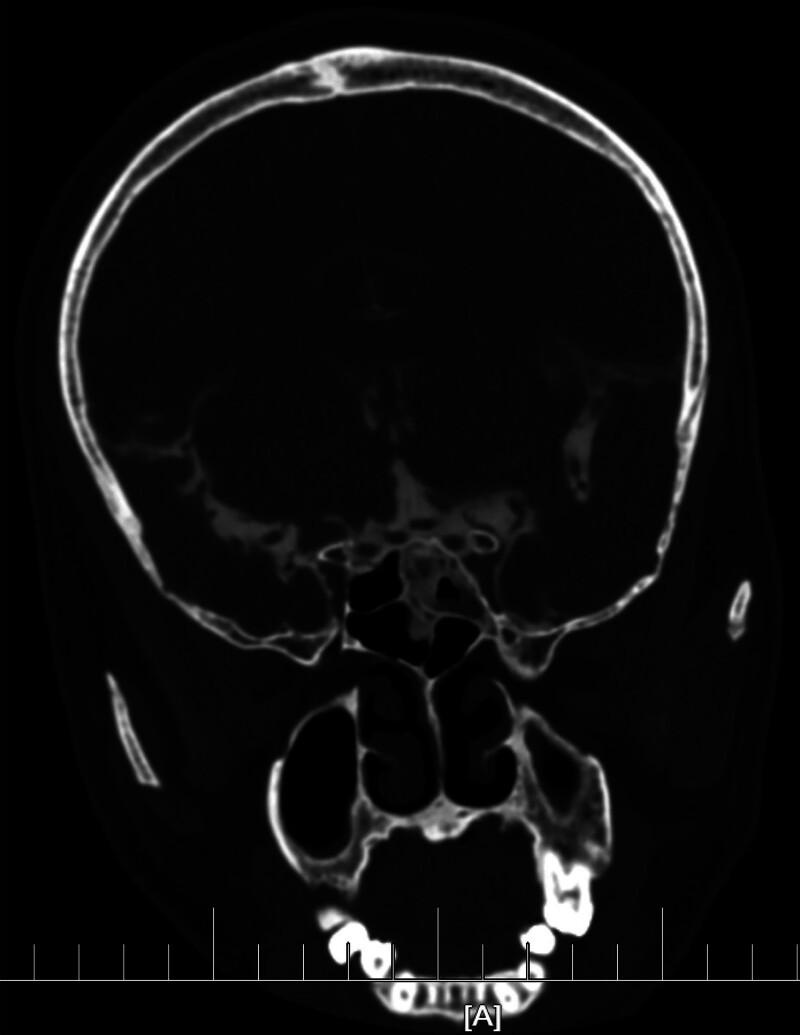
On the CT image of the patient on September 20th, ventricular angiography showed that the ventricular system, sulcus and cistern were filled with contrast agent, and the bone on the top wall of the right sphenoid sinus was discontinuous, with local bone defect shadow, and the contrast agent shadow was connected with the high density shadow of the right sphenoid sinus. CT = computed tomography.

After admission, the patient was treated with antibacterial, antiviral, dehydration and symptomatic support. The patient’s condition worsened rapidly. On September 4th, the patient developed irritability, confusion and dilated pupils. Considering the aggravation of central nervous system infection with cerebral hernia, the right lateral ventricle external drainage was performed urgently. Postoperative CSF culture suggested that Streptococcus pneumoniae was infected, and the patient’s condition was obviously improved after anti-infection treatment with sensitive antibacterial drug Ceftriaxone sodium. After repeated lumbar puncture, it was confirmed that the intracranial infection was under control. After cisternography was performed to find the leak (Figs. 2 and 3) and remove the contraindications, the CSF rhinorrhea was repaired by transsphenoidal endoscope on October 2. Operation method and postoperative treatment: The patient was taken in supine position. After general anesthesia was successful, a straight incision with a length of about 5 cm was marked on the lateral side of the right thigh. The skin in the operation field was disinfected routinely, covered with sterile towels, and subcutaneous adipose tissue and myofascia of appropriate size were cut for leak repair. The skin around the nose was routinely disinfected, covered with sterile towels, and the nasal mucosa was cut into a flap shape through the right nostril approach. The 0.1% epinephrine cotton piece was placed in the nasal cavity to shrink the mucosa and electrocoagulation to stop bleeding. The ostium of the affected sphenoid ethmoid sinus was found under the direct vision of nasal endoscope. The sphenoid sinus was opened with rongeur and high-speed drill, and the front wall and side wall of the sphenoid ethmoid sinus were bitten off to find the CSF leak. Scraping the mucosa of sinus cavity and mucosa around the leak to repair the wound, plugging the spare muscle fascia and subcutaneous adipose tissue into the dura mater inside the leak, filling the surgical cavity with gelatin sponge and hemostatic gauze, covering it with pedicled muscle mucosa flap of middle turbinate, and filling the right nasal cavity with oil yarn strip, and the operation is over. The operation was successful, and continuous LCD was performed after the operation to facilitate the healing of the leak, and anti-infection treatment was given at the same time. After comprehensive treatment, the patient’s condition was stable and his eyes were blurred. After removing the gauze packing in nasal cavity, the patient had no obvious discomfort or cerebrospinal fluid leakage, and was discharged.

## 3. Discussion

CSF rhinorrhea is one of the common complications in patients with craniocerebral trauma. CSF leakage can cause pathogenic bacteria to retrogress, and then lead to intracranial infection, which in turn leads to increased intracranial pressure, aggravated CSF leakage, and forms a vicious circle of repeated infection.^[13]^ Therefore, it is necessary to deal with intracranial infection first and perform CSF repair after infection control. This patient has CSF rhinorrhea with intracranial infection. During the treatment, lateral ventricle puncture and drainage were used to relieve intracranial pressure, and effective antibacterial drugs were used for active antibacterial treatment. After treatment, CSF rhinorrhea was repaired. The reliable means of leakage prevention is to use hemostatic gauze and gelatin sponge to fill the repair area, which plays the roles of water absorption and hemostasis of hemostatic gauze and solidification and plugging of gelatin sponge from different angles, and achieves comprehensive, ideal and reliable leakage prevention effect.^[14]^ It is difficult to repair the defect. At present, local pedicled skin flap or free skin flap is often used to cover the wound to protect the brain tissue, prevent CSF leakage and avoid retrograde intracranial infection.^[15]^ The local pedicled flap cannot meet the needs of large-scale wound repair because of its low tissue content. Therefore, it is more appropriate to repair with free flap. Free flaps commonly used for repair include anterolateral thigh flap, rectus abdominis myocutaneous flap, latissimus dorsi myocutaneous flap and forearm tissue fascial flap.^[16]^ Anterolateral thigh flap has become a common flap for repair because of its hidden donor site, rich tissue types, long vascular pedicle and simultaneous operation by 2 groups of doctors.^[17]^ CSF drainage often uses intermittent or continuous LCD to promote leakage healing.^[18]^ CSF drainage should be as uniform as possible to avoid excessive drainage. The basic drainage volume is 20 to 30 mL every 8 hours, and the flow speed and flow rate are adjusted according to the patient’s age, weight, ventricular size and drainage effect. At the same time, closely observe the condition, be alert to pneumocranium, brain displacement and even cerebral hernia coma caused by excessive drainage, and prevent bacteria from entering the skull through the drainage port.^[19]^ If serious complications occur, emergency surgery is needed to alleviate the life-threatening conditions such as pneumocranium and cerebral hernia. When there are contraindications of LCD, such as obstructive hydrocephalus, disappearance of cistern, etc, or LCD cannot be implemented, in order to avoid intracranial hypertension, we can consider taking extra-ventricular drainage or special long-distance extra-ventricular drainage as appropriate, but there is no relevant research on the treatment of CSF leakage by long-distance extra-ventricular drainage at present.^[20]^

High-flow CSF leakage needs repair surgery, and effective and firm skull base reconstruction has become the key to the success of the operation. According to the degree of skull base defect and CSF leakage, there are many materials and techniques for skull base reconstruction.^[21]^ Traditional skull base repair methods often use nasal mucosa flap to repair dura mater defect. The nasal mucosa flaps reported in the literature are mainly taken from nasal septum, middle turbinate, inferior turbinate and the lateral nasal cavity. Reconstruction of skull base with nasal septum mucosal flap with vascular pedicle, named Hadad–Bassagasteguy mucosal flap (HBF), significantly reduced the incidence of CSF leakage after operation, and became a recognized classic skull base repair technique.^[22]^ This technique can achieve effective, lasting and stable skull base reconstruction. Free middle turbinate mucosal flap was used for reconstruction of the sellar compartment. After operation, LCD was irregular, and the incidence of CSF leakage was 6.9%. However, the increased risk of CSF leakage during operation is related to the failure of reconstruction with avascular free graft. For patients with high CSF leakage during operation, the reconstruction of bone defect is the key to reduce the risk of CSF leakage after operation, and the skull base reconstruction can be successfully completed by combining either pedicled mucosal flap or free mucosal flap.

The advantages of pedicle nasoseptal flap are that it has the support of blood supply, the mucosa heals quickly, and the available mucosa area is large enough to repair the large skull base defect. However, the disadvantage of pedicle nasoseptal flap is that the vascular pedicle is prone to torsion during the repair process, which affects the blood supply of mucosal flap and leads to mucosal flap necrosis. The preparation of pedicle nasoseptal flap can cause extensive injury in the nasal cavity, and there may be hyposmia or loss of sense of smell, perforation of nasal septum, nasal adhesion and epistaxis after operation. During the operation, the tissue wounds around the skull base defect, especially the meninges and the surrounding bone windows, can be fully exposed, which can provide sufficient blood supply for the survival of the mucosal flap. Because the middle turbinate mucosa is rich in blood vessels, it is easy to survive as a graft. Complications related to mucosal flap include nasal scab, nasal bleeding, olfactory disturbance and so on.

In recent years, with the popularization of pedicled mucosal flap and Gasket–Seal Technology, the success rate of repairing CSF leakage with medium and high flow rate has been greatly improved, and the risk of CSF leakage has been reduced to 4.5%. Because of the bleeding of dural incision edge during operation, electrocoagulation can cause it to shrink and lead to dural defect, so it is difficult to achieve the effect of watertight suture.

The necessity and effectiveness of continuous drainage of lumbar cistern after operation are still controversial. Before 2015, LCD was usually performed, but there were some defects such as management difficulties, retrograde infection, venous thrombosis of lower limbs and prolonged bed rest. After the drainage of lumbar cistern, we must strictly observe the changes of the patient’s waist condition. If there is severe pain in the waist and numbness of the lower limbs, we must carry out corresponding tests in time to clarify the progress of the disease and treat it as soon as possible. Pay attention to disinfection and aseptic operation when dealing with drainage of lumbar cistern. Grasp the height of the drainage bottle to avoid excessive drainage.

The present case report has certain limitations, which should be mentioned. First, it only describes 1 case, and this disease is known to appear in a number of forms. In addition, the patient came to the hospital for reexamination regularly, but the follow-up period after discharge was only 6 years. Moreover, this study is limited by the lack of comparison between our surgical methods and other skull base reconstruction methods. In the future, large-sample and multi-center prospective research is needed to verify the conclusion of this study.

## Author contributions

**Data curation:** Jiatao Lv.

**Funding acquisition:** Longshan Zhou, Xiaoqian Yang, Jiatao Lv.

**Project administration:** Longshan Zhou, Zonglei Chong.

**Resources:** Longshan Zhou, Xiaoqian Yang, Jiatao Lv.

**Supervision:** Xiaoqian Yang.

**Writing – original draft:** Zonglei Chong.

**Writing – review & editing:** Zonglei Chong.
